# Estimating prognostic relevant cutoff values for a multiplex PCR detecting BCR::ABL1 in chronic myeloid leukemia patients on tyrosine kinase inhibitor therapy in resource-limited settings

**DOI:** 10.1007/s00277-023-05254-x

**Published:** 2023-05-22

**Authors:** Saifu Hailu, Samuel Kinde, Michael Cross, Aster Tsegaye, Tsehayneh Kelemu, Daniel Seifu, Dawit Alemayehu, Azeb Tarekegn, Gutema Jabessa, Desalegn Abeje, Markos Abebe, Abdulaziz Sherif, Fisihatsion Tadesse, Uwe Platzbecker, Rawleigh Howe, Amha Gebremedhin

**Affiliations:** 1 College of Health Sciences, Addis Ababa University, Addis Ababa, Ethiopia; 2 Leipzig University Hospital, Leipzig, Germany; 3 Armauer Hansen Research Institute, Addis Ababa, Ethiopia; 4 Madda Walabu University, Bale Robe, Ethiopia

**Keywords:** Chronic myeloid leukemia (CML), Multiplex PCR, Prognostic relevant cutoff values, BCR::ABL1 fusion gene, Low- and middle-income countries (LMICs), Clinical lab performance

## Abstract

The prognosis of chronic myeloid leukemia (CML) on tyrosine kinase inhibitor (TKI) treatment is based on the quantification of BCR::ABL1 fusion gene transcript copy number, harmonized by an international scale (IS) based on TaqMan-based real-time quantitative PCR (qRT-PCR). In Ethiopia, as in most low- and middle-income countries (LMICs), access to standard diagnostic, follow-up, and prognostic tools is very limited, and it has been challenging to strictly follow international guidelines. This seriously compromises clinical outcome, despite the availability of TKIs through the Glivec International Patient Assistance Program (GIPAP). Multiplex PCR (mpx-PCR), conventionally regarded as a “screening tool,” offers a potential solution to this problem. A total of 219 samples from confirmed CML patients were assayed. In reference to qRT-PCR, the AUC of ROC curve for mpx-PCR was 0.983 (95% CI: 0.957 to 0.997). At the optimum cut-off value, equivalent to BCR::ABL1 (IS) transcript copy number of 0.6%, the specificity and sensitivity were 93% and 95%, respectively, with 94% accuracy. Albeit the sensitivity and accuracy of mpx-PCR decrease below the optimum cutoff of 0.6% (IS), the specificity at 0.1% (IS) was 100%, making it an attractive means to rule-out relapse and drug non-adherence at later stages of treatment, which is particularly an issue in a low income setting. We conclude that the relative simplicity and low cost of mpx-PCR and prognostic relevant cutoff values (0.1–0.6% IS) should allow its use in peripheral clinics and thus maximize the positive impact of TKIs made available through GIPAP in most LMICs.

## Background

The hallmark of chronic myeloid leukemia (CML) is the Philadelphia chromosome (Ph), which occurs in more than 95% of patients. This chromosomal alteration involves the reciprocal translocation of the long arms of chromosome 22 at the BCR gene and chromosome 9 at the ABL gene (t[9;22]), which is responsible for the constitutively deregulated tyrosine kinase activity and inhibition of apoptosis [1-3].

The introduction of tyrosine kinase inhibitors (TKIs) in the treatment of CML has drastically transformed survival, giving patients with chronic-phase (CP) disease a near-normal age-adjusted lifespan [4]. Recent evidence showed that in selected patients with sustained deep molecular response, treatment-free remission (TFR) has become a feasible and safe option [5].

Nevertheless, in low- and middle-income countries (LMICs), due to the fact that access to standard diagnostic, follow-up, and prognostic tools is very limited [6], it has been challenging to adhere strictly to international guidelines. This seriously compromises clinical outcome, despite the availability of TKIs.

In Ethiopia for example, more than 80% of CML patients with poor clinical outcome are not verified by molecular analysis [7]. TKI guidance or relapse risk most often has to be assessed on the basis of hematological rather than molecular parameters due to limited access to molecular test services, even in the tertiary care referral hospitals. Although hematological parameters clearly play an indispensible role, the quantification of BCR::ABL1 levels on the normalized-international scale (IS) using methods based on quantitative real time PCR (qRT-PCR) is essential to guide treatment decisions at milestone time points and is a mandatory to the consensus guidelines established by the European Leukemia Network (ELN) [8] and the US National Comprehensive Cancer Network (NCCN) [9].

Sensitive, accurate, and reliable quantification of BCR::ABL1 is a cost-effective outcome prediction and a prerequisite for achieving the goals of treatment-free remission (TFR) [10], and for the effective management of therapy to enable patients to take advantage of the growing spectrum of “targeted” therapeutics entering the market.

Improving access to, affordability of, and outcomes of cancer treatment has been identified as one of the five priorities for cancer research in LMICs [11]. No-cost access to TKIs in LMIC has been provided by the Glivec® International Patient Assistance Program (GIPAP) launched by Max Access Solutions (MAS) in partnership with Novartis. However, for the reasons explained above, candidate institutions need to have a basic diagnostic platform in order to qualify [12]. In an attempt to bridge this gap, the MAS provides a BCR:ABL monitoring point-of-care assay [13], the Cepheid GeneXpert®, which increases access to tests in more than 60 LMICs [14] including Ethiopia. However, even at the preferential (below market) price of $50, single-test cartridges still represent a forbiddingly high cost in most LMIC.

Multiplex PCR (mpx-PCR), conventionally regarded to be a “screening tool,” offers a potential solution to this problem. Mpx-PCR is considerably less cumbersome and less costly than qRT-PCR, but retains the capacity to detect both typical and atypical BCR::ABL1 fusion transcripts down to a frequency of 10^−3^ [15, 16].

In order to evaluate the potential of mpx-PCR as a viable alternative to qRT-PCR in the management of TKI therapy in LMIC, we have compared these techniques in terms of prognostic benefit in predicting risk of relapse among CML patients on TKI treatment.

## Method

### Study setting and population

Recruitment of CML patients was conducted during regular clinic visits for follow-up or drug refill schedule at the hematology clinics of Tikur Anbessa Specialized Hospital (TASH), a tertiary care teaching and referral hospital in Addis Ababa, Ethiopia.

Consenting, cytogenetically confirmed Ph+ CML patients visiting the hematology clinic were enrolled consecutively. Five-milliliter (ml) peripheral blood (PB) was collected in Vacutainer EDTA Tubes (Becton Dickinson) from a total of *N* = 219 consenting adult patients. Study participants were those who were treatment naïve or on first line or subsequent TKI therapy. Clinical data was extracted from the clinical record.

### Sample processing

All samples were processed within 2 h of collection. Briefly, samples were mixed with four-fold ice-cold NH_4_Cl lysing buffer (pH 7.4), kept for 20–25 min on ice until RBC lysis was apparently complete. The preparation was washed twice with ice-cold PBS by centrifugation for 5 min at 300 g. Pelleted white blood cells were counted using a hemocytometer after staining with 0.4% Trypan Blue (Sigma Aldrich) and adjusted to 10 million cells per vial. The pellets were then completely lysed in 600 μL of guanidine thiocyanate buffer (GTC) containing 8 μL/ml β-mercaptoethanol. Duplicate vials of the GTC lysate were stored at − 80 °C.

### RNA extraction and cDNA synthesis

The RNeasy Mini Kit (QIAGEN #74106) was used to extract total RNA according to the manufacturer’s protocol. Briefly, frozen lysates were thawed in a 37 °C water bath and put through a QIAshredder spin column (QIAGEN #79656) and then into RNeasy column for extraction of total RNA. The purity and quantity of eluted total RNA was measured using a Thermo Scientific™ NanoDrop™ One Spectrophotometer. cDNA was synthesized using Invitrogen™ SuperScript™ IV VILO™ Master Mix according to the manufacturer’s instructions.

### Multiplex PCR

The mpx-PCR procedure was adopted based on a method described [17]. Four primers (Table 1) were used for amplification of various targets of the BCR::ABL1 fusion gene.

**Table 1:** Primer set used for multiplex PCR [17]

Primers	Primer sequences	Primer binding site on BCR::ABL1
(1)	5′-ACCGCATGTTCCGGGACAAAAG-3′	BCR Exon 1 (forward-1),
(2)	5′-ACAGAATTCCGCTGACCATCAATAAG-3′	BCR Exon 13 (forward-2),
(3)	5′-ATAGGATCCTTTGCAACCGGGTCTGAA-3′	BCR Exon 21 (reverse-1),
(4)	5′-TGTTGACTGGCGTGATGTAGTTGCTTGG-3′	ABL1 Exon 3 (reverse-2).

This primer set amplifies all possible BCR::ABL1 transcript variants: M-BCR::ABL1 (either b2/a2 or b3/a2 with 310 bp or 385 bp, respectively), m-BCR::ABL1 (e1/a2 with 481 bp), μ-BCR::ABL1 (e19a2 with 927 bp), and rare transcripts (e6a2 with 1125 bp and e8a2 with 1319 bp). As an internal control for the overall amplification process, the primer set amplifies wild-type BCR transcript to generate a fragment of 808 bp.

The PCR reaction mixture was prepared in a total of volume of 23 μL with a final concentration of 2.5 mM MgCl_2_, 0.25 μM of each primers, 1 Unit Taq polymerase, 1× PCR buffer with 0.2mM dNTPs, and 2 μL template cDNA.

The thermo-cycler program was 10 s 100 °C, 1 min 96 °C, 3 min 58 °C, 2 min 72 °C (10 s 100 °C, 20 s 97 °C, 25 s 56 °C, 25 s 58 °C, 10 s 78 °C, 90 s 73 °C) 31×, 10 min 73 °C, then 4 °C.

The following cell lines were used to derive control cDNA: K562 cells as M-BCR::ABL1 controls (b3/a2) 385bp, BV173 cells as M-BCR::ABL1 controls (b2/a2) 310bp, SD-1 cells as m-BCR::ABL1 control (e1/a2) 481bp. Non-template controls (multiplex BCR::ABL1 PCR mix alone) and a negative control including the transcription mix without RNA (RT-Mix + BCR::ABL1 PCR mix) were included in every run.

PCR products and 100bp ladder were loaded into 2% agarose gel (Sigma Aldrich) in tris-acetate EDTA (TAE) buffer and GelRed® Nucleic Acid Stain (Sigma Aldrich), which was run for 35 min at 4 volts/cm. Printout image was documented after automatic image resolution.

### qRT-PCR

The TaqMan-based real-time quantitative PCR (qRT-PCR) protocol was as described earlier [18, 19] with the following primer/probe set for BCR::ABL1 and ABL1 (Table 2).

**Table 2:** Primer and probe set for qRT-PCR

Target amplification	Primer/probe set:
M-BCR::ABL1	5′- TCCGCTGACCATCAAYAAGGA-3′ (forward)
	5′-CACTCAGACCCTGAGGCTCAA-3′ (reverse)
	5′-CCCTTCAGCGGCCAGTAGCATCTGA-3′ (probe)
ABL1	5′-TGGAGATAA-CACTCTAAGCATAACTAAAGGT-3′ (forward)
	5′-GATGTAGTTGCTTGGGACCCA-3′ (reverse)
	5′-CCATTTTTGGTTTGGGCTTCACACCATT-3′ (probe)

The reactions were set up using TaqMan™ Universal PCR Master Mix, No AmpErase™ UNG (Applied Biosystems #4324018).

Calibration [20] of qRT-PCR was done using a set of six plasmid certified reference materials (CRM) (ERM ®  — AD623a − f) [https://joint-research-centre.ec.europa.eu/reference-measurement_en]. Briefly, two sets of plasmid CRM (BCR::ABL1 (e14a2) and control gene ABL1) with known copy number concentration were run in triplicate. Calibration curves were plotted using cycle threshold (*C*_*T*_) vs. copy number concentration/μl (10 to 10^6^) for each sets of plasmid CRM. The calibration curve was also used to monitor the amplification efficiency of each experiment.

Using *C*_*T*_, the actual copy number of BCR::ABL1 and ABL1 (housekeeping gene) for the unknown samples was extrapolated from the calibration curves. Each sample was analyzed in duplicate. The normalized percent international scale (BCR::ABL1 %IS) was computed by the ratio of copy number of BCR::ABL1 to ABL1, multiplied by a laboratory-specific conversion factor of 0.542 (adopted during the European collaborative harmonization study—European Treatment and Outcome Study (EUTOS)) [21] and then converted to percentage:

$$BCR::ABL1\% IS=\frac{Sum\ of\ copy\ number\ of\ Bcr::Abl1\ from\ sample}{Sum\ of\ copy\ number\ of\ Abl1\ from\ sample} \times 100\times 0.5$$

### Data analysis and interpretation [22]

While the mpx-PCR yields a single binary result (positive or negative), qRT-PCR yields a continuous number that reflects the frequency of target sequences in the original sample. To compare the performance of the methods, the qRT-PCR results were converted to binary positive/negative values around all possible cutoff values. Then, a receiver operating characteristic (ROC) curve was computed by plotting pairs of specificity and sensitivity across all possible cutoff values based on a nonparametric application of DeLong’s test (StataCorp. 2013. Stata Statistical Software: Release 13. College Station, TX: StataCorp LP). Sensitivity was defined as the proportion of mpx-PCR-positive samples among those for which qRT-PCR values exceeded the cutoff, while specificity was defined as the proportion of mpx-PCR-negative cases among those determined by qRT-PCR to be below cutoff.

The ability of mpx-PCR to reproduce the results of qRT-PCR was then further plotted based on sensitivity, specificity, and accuracy at each of 10 chosen cut-off values, covering the range from 0.1 to 1% (IS).

## Result

### Study participants’ profile

Of the total of 219 CML patients enrolled in the study, valid assay results for mpx-PCR were obtained from 201 samples. The median age of the CML patients was 35 (range: 19–83) and the duration of treatment ranged from 0 to144 months with 63% patients on treatment for more than 1 year. Of the participants, 139 were male and 80 female (M/F ratio of 1.7:1). Among total participants, 49 (22.4%) were treatment naïve (Table 3).

**Table 3:** The study participants’ treatment duration

Treatment-naïve patient	Patients on TKI treatment for a certain duration				
	1–3 months	3–6 months	6–12 months	> 1 years	Total
49 (22.4%)	16 (7.3%)	5 (2.2%)	11 (5.0%)	138 (63.0%)	219

### Estimating prognostically relevant cutoff values

**Fig. 1:**
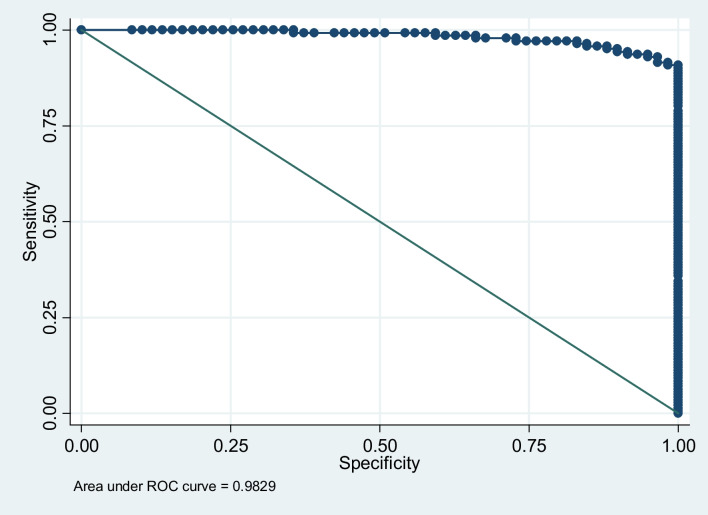
Receiver operating characteristic (ROC) curve of specificity vs sensitivity across all possible cutoff points; AUC − 0.98 (95%CI: 0.960 to 0.997)

Based on a comparison of mpx-PCR vs the “gold standard” qRT-PCR, the AUC of the receiver operating characteristic (ROC) curve, which was plotted based on specificity vs. sensitivity across all possible cutoffs, was 0.983 (95% CI: 0.957 to 0.997) (Fig.1).

**Fig. 2:**
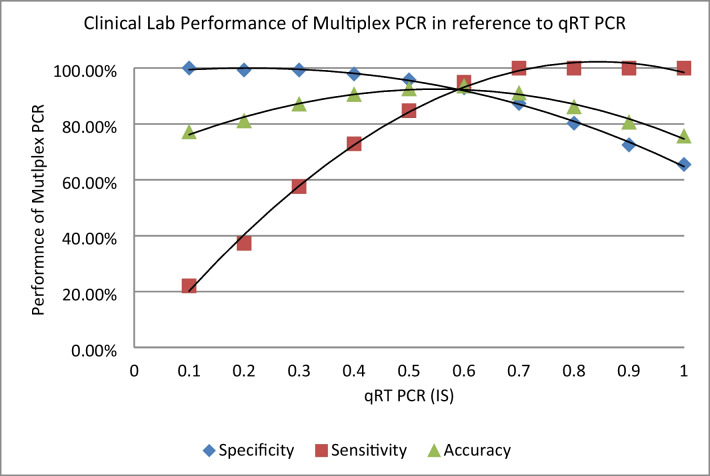
The specificity, sensitivity, and accuracy by mpx-PCR compared to qRT-PCR at different cutoff values. At 0.6% (IS), the specificity and sensitivity were 93% and 95%, respectively, with 94% of accuracy

Indicators of clinical lab performance such as sensitivity, specificity, and accuracy of mpx PCR in comparison to the gold standard qRT-PCR are further depicted in Fig. 2.

The sensitivity describes the proportion of qRT-PCR positive samples that are also scored positive by mpx-PCR. At qRT-PCR cut-off values of 0.7% and above (corresponding to high frequencies of BCR::ABL1 transcript), this value is 100%. The sensitivity of mpx-PCR was still 94.92% using a cut-off of 0.6%, but dropped sharply thereafter, as the mpx-PCR technique failed to detect some of the low-level samples.

The specificity is expressed as the proportion of samples below the qRT-PCR cut-off that also scored negative by mpx-PCR. Specificity at very low target frequencies is 100%, as expected from a stringent PCR in the absence of contamination. It is important to note that the resulting decrease in specificity and accuracy at high cut-off values (corresponding to higher target frequencies) is due to mpx-PCR generating positive signals from samples with BCR::ABL1/ABL1 frequencies below the chosen cut-off and not from those that lack BCR::ABL1 entirely (Table 4).

**Table 4:** BCR::ABL1 transcript copy number (IS) of the study participants

%IS	< 0.001	0.001–0.1%	0.11–1%	1–10%	> 10%	Total
Patients, *N* (%)	9 (4.1%)	35 (16%)	25 (11.4%)	24 (11%)	126 (57.5%)	219

As summarized in Fig. 2, this shows that mpx-PCR is sufficiently sensitive to detect a BCR::ABL1 transcript frequency of 0.6% (IS) or more with high reliability.

## Discussion

The prognosis of chronic myeloid leukemia (CML) has improved drastically following the introduction of TKI therapy both in high-income countries (HICs) and, enabled by GIPAP, also in LMICs [23, 24].

However, the impact in LMICs has been limited by the necessity for reliable, quantitative molecular diagnostics in order to guide TKI therapy. This is likely to contribute to the increased death mainly observed in lower social-demographic index (SDI) countries [25]. In 2019, the highest age-standardized death rate was observed in Ethiopia (1.89 per 100,000) [26]. Although deaths associated with CML were predominantly among patients above the age of 70 years, the overall incidence rate was skewed towards the younger population (median age at the diagnosis was 33) most of whom presented with features of advanced-phase disease [7]. Despite the availability of the GIPAP, there has been limited access for the mandatory standard molecular prognostic tools to regularly monitor CML patients on TKI treatment.

According to the NCCN® clinical practice guidelines on CML [9], the most important goal of first-line TKI therapy is to prevent disease progression through regular surveillance (every 3–6 months), following achievement of the treatment milestone of BCR::ABL1(IS) ≤ 1% at 12 months,. However, regular monitoring using the standard qRT-PCR protocol is often impractical in the limited setting and facilities typical of LMICs. For this reason, context-specific prognostic tools are key to enabling guidance of TKI therapy, and the development or adaptation of such tools to a resource limited setting is a major priority for LMICs [11].

Previous studies [15, 16] have demonstrated that mpx-PCR, though essentially a qualitative assay for detecting and distinguishing various BCR-ABL1 transcripts, has an analytical sensitivity down to 10^−3^. Since mpx-PCR would be a relatively simple, low-cost, and rapid means to screen disease status in CML patients receiving TKI treatment, we evaluated the clinical lab performance of mpx-PCR in reference to qRT-PCR harmonized international scale (IS), and its potential prognostic value in our limited setting.

We found the overall clinical lab performance of mpx-PCR to be comparable with qRT-PCR calibrated against certified plasmid reference material, with an AUC of ROC curve of 0.983 (95% CI: 0.957 to 0.997) (Fig. 1). At the optimum cut-off value, i.e., equivalent to a BCR::ABL1 (IS) transcript copy number of 0.6%, the specificity, sensitivity, and accuracy were 93.0%, 95%, and 93.5%, respectively (Fig. 2). In first-line therapy, the reduction of BCR::ABL1 copy number to between 0.1 and 1%(IS) during the first 6 to 12 months after TKI treatment is a major treatment milestone [9], with these patients having a lower risk of disease progression and relapse [27, 28]. In this context, the specificity and sensitivity of mpx-PCR demonstrated here would be sufficient to guide therapy and have a significant clinical benefit. Albeit the sensitivity and accuracy of mpx-PCR decrease below the optimum cutoff of 0.6%, the specificity at 0.1% was 100%, making it an attractive means to rule-out relapse and drug non-adherence at later stages of treatment, which is a particularly important issue in managing patients in a rural, low-income setting [29]. Also, as most of our CML patients are young (median 35, data not shown), we are often faced with the issue of treatment discontinuation during pregnancy and fertility planning. This is a process that could definitely be addressed by monitoring using mpx-PCR. It should be noted that the performance of mpx-PCR assessed here was based on duplicate assays in order to limit costs. Depending on resource availability, it should be feasible to increase sensitivity further by preforming triplicate assays.

Finally, the availability of a relatively low-cost and simple technique for the sensitive detection of BCR::ABL1 in TKI-treated patients would allow us to investigate the potential for achieving treatment-free remission (TFR) in patients with good drug adherence and response. To the best of our knowledge, this issue has not yet been approached in a sub-Saharan African CML population.

We conclude that the relative simplicity and low cost of mpx-PCR and prognostic relevant cutoff values (0.1–0.6% IS) of mpx-PCR is sufficient to justify its use in peripheral clinics and thus maximize the positive impact of TKIs made available through GIPAP in most LMICs

### Study limitation

Our study was performed on peripheral blood samples due to the relative ease of collection. If feasible, it would be worth extending the study to aspirated bone marrow samples.
